# 

**Published:** 1891-03

**Authors:** 


					﻿MARCH, 18©1.
OLD SERIES
VoU XXV
NEW SERitfS 1
Vol. VIII.—No. I. /
4* 1855 v
IHtwwb

JnilBNAk
■wPjiore»,


H. V. M. MILLER, M. D., LL.D.
VIRGIL 0. HARDON, M. D.
F. W. McRAE, M'. D.
L.	P. KENNEDT^ M., M. D.
M.	B. HUTCHINB. M. D.
L. B. GRANDTL M\ D.
MuBUSHEDBY JAMES RHARRISON&C®
Mg_____________tST LANTA, GA.
subscription t 12.00 a wail
				

